# Perceptions of the quality of the therapeutic alliance in chiropractic care in The Netherlands: a cross-sectional survey

**DOI:** 10.1186/s12998-016-0100-4

**Published:** 2016-06-08

**Authors:** Nicoline M. Lambers, Jennifer E. Bolton

**Affiliations:** Anglo-European College of Chiropractic, 13-15 Parkwood Road, Bournemouth, BH5 2DF UK

**Keywords:** Therapeutic alliance, Working alliance, Working relationship, Chiropractic, Doctor-patient relationship

## Abstract

**Background:**

Research in various medical fields demonstrates a consistent and positive association between clinical outcomes and the quality of the therapeutic alliance between the patient and clinician. The aim of this study was to explore how well chiropractors and their patients in The Netherlands perceive the quality of their working relationship.

**Methods:**

A nationwide survey of chiropractors and their patients was conducted in The Netherlands, using a validated Dutch translation of the Working Alliance Inventory (WAV-12). Data were collected over a 5-week period in September-October 2014. Both patients and chiropractors were requested to reflect on 12 statements about to how well they perceived their collaboration in reaching consensus on treatment goals and treatment strategies, and how well they perceived the existence of an affective bond in their working relationship. A 5-point Likert scale was used to answer each question. Higher ratings reflected a more positive perception of the therapeutic alliance. Furthermore, levels of agreement between patients’ and chiropractors’ perceptions of the quality of their therapeutic alliance were determined.

**Results:**

In total, 207 working relationships between patients and their chiropractor were analysed. The quality of the therapeutic alliance was perceived as being very positive for both patients (*n* = 183, mean 49.14 ± 7.12) and chiropractors (*n* = 202, mean 50.48 ± 4.97). There was no difference in patients’ perceptions whether treated by a male or female chiropractor, nor in relation to the chiropractor’s years of experience. Nevertheless, poor agreement was found between perceptions of patients and chiropractors in the same relationship (ICC = 0.13).

**Conclusions:**

Both patients and chiropractors perceived the quality of the therapeutic alliance as being very positive. Despite these positive results, patient and chiropractor pairs perceived the level of collaboration in order to reach agreement on treatment goals and strategies and the quality of their affective bond very differently. Clinically, these results suggest that chiropractors should, during the course of treatment, continue to collaborate with their patient and frequently verify whether their patient continues to agree with the treatment goals and treatment plan applied to further develop, improve and maintain a positive therapeutic alliance.

**Electronic supplementary material:**

The online version of this article (doi:10.1186/s12998-016-0100-4) contains supplementary material, which is available to authorized users.

## Background

With regard to improving the quality of care, a paradigm shift is currently advocated in medicine from disease-centred care towards patient-centred care. One of the fundamental requirements for practising patient-centred care is developing a therapeutic working relationship with the patient [1]. In the literature, this therapeutic working relationship is interchangeably referred to as therapeutic alliance, working alliance or helping alliance [1–4].

There is consensus about the three essential elements of the alliance based on Bordin’s concept of the Working Alliance [5]. The first two elements relate to the collaboration between the patient and clinician in reaching agreement on the treatment goals (goal dimension) and treatment strategies, or tasks, applied to achieve the goals (task dimension). The third element is the presence of an affective bond between the patient and clinician. An affective, emotional connection such as, for example, mutual trust and acceptance will favour collaboration and reaching consensus on treatment goals and treatment strategies [2–4, 6, 7].

The concept of the therapeutic alliance has become increasingly significant due to its positive and consistent association with treatment outcomes. This association was first identified in psychotherapy research [2–4] and more recently in research in the fields of general medicine [8–12] and physical therapy and rehabilitation [13–15]. To improve the quality of the therapeutic alliance, it is imperative for a clinician to understand which factors influence and enhance the quality of the therapeutic alliance.

**Fig. 1:**
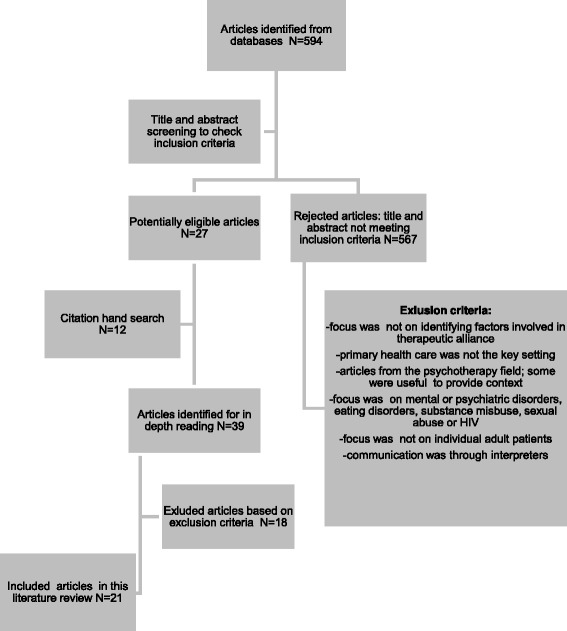
Selection process of articles for literature review

A literature search (Additional file 1 and Fig. 1) was performed to identify the core components of the therapeutic alliance that determine its quality in relation to clinical outcomes in primary care settings. These were found to be: (1) empathy, (2) trust, (3) collaboration, (4) agreement on treatment goals and treatment strategies, and (5) patient-centred communication. Most research related to the association between empathy and trust with treatment outcomes, indicating the apparent relevance of the development of an affective bond as part of the alliance.

Empathy is a fundamental and crucial factor in the therapeutic alliance, and it is highly valued in relation to quality of care by both patients and clinicians [9, 16–19]. A recent systematic review found that an empathic behaviour on the clinician’s side diminishes anxiety and distress in patients and improves patient satisfaction, patient adherence and patient enablement in medical settings [9].

Trust also influences the affective bond and is defined as ‘the belief that the doctor is working in the patient’s best interest’. It is found to be associated with increased patient satisfaction, adherence to treatment and continuity of care [12, 20–22]. Surprisingly to some, clinicians’ behaviours that foster trust as perceived by patients are, above all, being comforting and caring, indicating that the affective characteristics of the clinician apparently provide the patient with more trust than do the technical competencies of the clinician [22].

Collaboration, or partnership, is considered another fundamental component of the therapeutic alliance [8, 23, 24] and is consistently associated with improved patient adherence and satisfaction with care [8, 25].

Although little research has been performed on the agreement on treatment goals and strategies component of the therapeutic alliance in primary care settings, it is also considered to be a fundamental part of the alliance [10, 11]. Positive outcomes are not very likely if there is no agreement on treatment goals and strategies between the patient and the clinician. Particularly in the case of chronic conditions, where self-management and self-efficacy are required from patients, consensus on treatment goals and strategies between the patient and the clinician have been found to improve treatment outcomes [26].

Finally, patient-centred communication is considered an essential and crucial component in the therapeutic alliance [11, 23, 27, 28], especially since it mediates the other core components of the alliance. Communication styles that help clinicians engage more with patients, and which facilitate patient participation, are associated with stronger therapeutic alliances [11].

Both understanding the concept of the therapeutic alliance and implementing the core components in clinical practice are essential in order to improve the quality of the working relationship. However, with respect to improving quality, it is important to know how patients and clinicians perceive the quality of the therapeutic alliance. Few studies have investigated this aspect, in particular in chiropractic care. The purpose of this study is, therefore, to describe the quality of the therapeutic alliance as perceived both by patients and by chiropractors in The Netherlands, and to determine whether patients and their chiropractors have similar perceptions of their encounter.

## Methods

### Participants

Participants were chiropractors working in The Netherlands and three of their adult patients. All members of the Netherlands Chiropractors’ Association (NCA) who worked in private practice in The Netherlands (*n* = 252) were invited to participate in this study by email. Patients that were eligible to partake in this study were the participating chiropractor’s first consecutive three patients after receiving the questionnaires who: (1) consulted their chiropractor for their third visit irrespective of their symptoms, (2) were over 18 years old, (3) were able to read and understand Dutch and (4) had not consulted the same chiropractor in the last 3 years, except for the first and second consultation. In the case of an eligible patient refusing to participate, the chiropractor was requested to recruit the next eligible patient fulfilling the inclusion criteria.

### Questionnaires

The questionnaires used to collect data for this survey were the client and therapist versions of the “Werkalliantievragenlijst (WAV-12) [29]. This WAV-12 is a translated (English to Dutch), shortened and revised version of the Working Alliance Inventory (WAI) [7, 30, 31]. This WAI is a self-report instrument based on Bordin’s concept of the Working Alliance [5] to assess the quality of the working relationship as perceived by the client and the therapist. It is one of the most frequently used alliance measures, both in psychotherapy and other medical fields [15, 29, 32–34].

The WAV-12 client version has demonstrated good internal consistency reliability (α = 0.82-0.85) and good construct validity (Goodness-of-Fit index 0.90) ([29]. Permission to use the WAV-12 for this study was received from Professor Horvath, the developer of the original WAI [30]. For this study, both the client and therapist versions of the questionnaire were slightly modified for use in a chiropractic setting by replacing the word client by patient, therapist by chiropractor, therapy by chiropractic and session by treatment.

### Data collection

All chiropractors that agreed to enrol were sent an envelope with three chiropractor versions and three patient versions of the questionnaire in. For each patient-chiropractor encounter, the patient and the chiropractor each completed one questionnaire, resulting in “paired questionnaires”. Both versions of the questionnaire consisted of twelve questions in the form of statements, reflecting patients’ experiences with respect to collaboration in reaching agreement on treatment goals (goal dimension), agreement on treatment strategies (task dimension) and on the existence of an affective bond (bond dimension) in the working relationship, with four statements for each area. Patients and chiropractors were asked to rate how often they felt each statement to be true in their working relationship on a 5-point Likert scale ranging from (1) seldom or never, (2) sometimes, (3) fairly often, (4) very often, to (5) always. In addition, all patients and chiropractors were asked for their gender. Chiropractors were also asked to note their number of years of working experience as a chiropractor.

All participants were requested to complete their questionnaires immediately after the patient’s third consultation. Research has shown that statistically reliable associations exist between the working alliance and clinical outcomes after the third consultation. Furthermore, the quality of the alliance early in the treatment was found to be a better predictor of outcomes than measured at later phases in the treatment [3, 30, 31]. In addition, as pointed out by Ferreira at al. (2013), assessing the alliance early in the treatment plan limits the influence of the clinical effect of the intervention [13]. Directly upon completion, all participants were instructed to put their questionnaire in an unmarked envelope, and personally seal it in order to safeguard anonymity and prevent anybody but the researcher from reading the answers. Data were collected over a five-week period in September-October 2014.

### Data analysis

Variables retrieved from the questionnaires were the patients’ and chiropractors’ ratings (1–5) for each of the twelve questions, their gender and the chiropractors’ years of working experience as a chiropractor. Data were analysed using SPSS (Version 20). To describe the overall quality of the working relationship, and the quality of the three separate elements (goal dimension, task dimension, bond dimension), as perceived by patients and chiropractors, descriptive statistics were used. Higher ratings indicated more positive perceptions of the alliance. The Intraclass Correlation Coefficient (ICC) was used to determine the level of agreement between patients’ and chiropractors’ perspectives of the alliance. The unpaired t-test was used to determine whether there was a difference in patients’ perspective dependent on chiropractors’ gender, and dependent on gender matching between the patient and their chiropractor. Finally, a one-way ANOVA test was used to determine the effect of the chiropractor’s years of working experience on patients’ perceptions.

## Results

### Participants

A total of 89 chiropractors (35.3 % of the invited NCA members) agreed to participate in this study. Of the 76 chiropractors (52.6 % female) that returned questionnaires, 60 (78.9 %) returned questionnaires on three working relationships, 12 (15.8 %) on two working relationships, and 4 (5.3 %) on one working relationship. One questionnaire was incomplete and excluded. Two reminders to participate were sent to all invited chiropractors and a further two emails were sent to remind chiropractors of returning their questionnaires. In total, data on 207 patient-chiropractor working relationships were included.

### Demographic characteristics

Demographic characteristics of the participants are shown in Table 1.

**Table 1:** Demographic characteristics of the participants

Variable	Characteristic	N	%
Gender (Chiropractors)	Male	36	47.4
	Female	40	52.6
	Missing data	0	0
	Total	76	100
Gender (Patients)	Male	84	40.6
	Female	118	57.0
	Missing data	5	2.4
	Total	207	100
Gender matching in	Matched	133	64.3
chiropractor-patient pairs	Unmatched	69	33.3
	Missing data	5	2.4
	Total	207	100
Years of working	0–5 years	23	30.3
experience (Chiropractors)	6–14 years	28	36.8
	15+ years	25	32.9
	Total	76	100

### WAV-12 scores and frequencies, overall and per dimension

As seen in Table 2, both patients (*n* = 183; mean 4.09 ± 0.59) and chiropractors (*n* = 202; mean 4.21 ± 0.41) rated their alliance very positive.

**Table 2:** Means of total scores and mean scores of patients and chiropractors on the WAV-12 overall and per dimension

Patient scores	N	Mean of total scores^a^	SD	Mean scores^b^	SD
Overall WAV-12	183	49.14	7.12	4.09	±0.59
Goal dimension	197	15.92	2.61	3.98	±0.65
Task dimension	201	16.39	2.46	4.10	±0.62
Bond dimension	188	16.89	2.80	4.22	±0.70
Chiropractor scores					
Overall WAV-12	202	50.48	4.97	4.21	±0.41
Goal dimension	206	16.18	2.21	4.04	±0.55
Task dimension	205	16.59	2.04	4.15	±0.51
Bond dimension	205	17.71	1.64	4.43	±0.41

^a^Overall WAV scores max 60; dimensional scores max 20

^b^Measured on a 1–5 point Likert scale, 5 representing an optimal alliance

Compared to patients’ perceptions, chiropractors perceived the quality of the working relationship slightly more positive, both on the quality of the therapeutic alliance overall, as well as on agreement on treatment goals and strategies and the presence of an affective bond separately. Both groups experienced the affective aspect within the alliance slightly more positive than the collaborative aspects. Agreement on goals, although still rated very positive, was perceived the least strong of the three dimensions. Fewer patients (*n* = 183, 88.4 %) than chiropractors (*n* = 202, 97.6 %) completed all twelve questions. Particularly questions exploring views on the quality of the affective bond (9.2 %) and agreement on treatment goals (4.8 %) were left unanswered by patients.

**Fig. 2:**
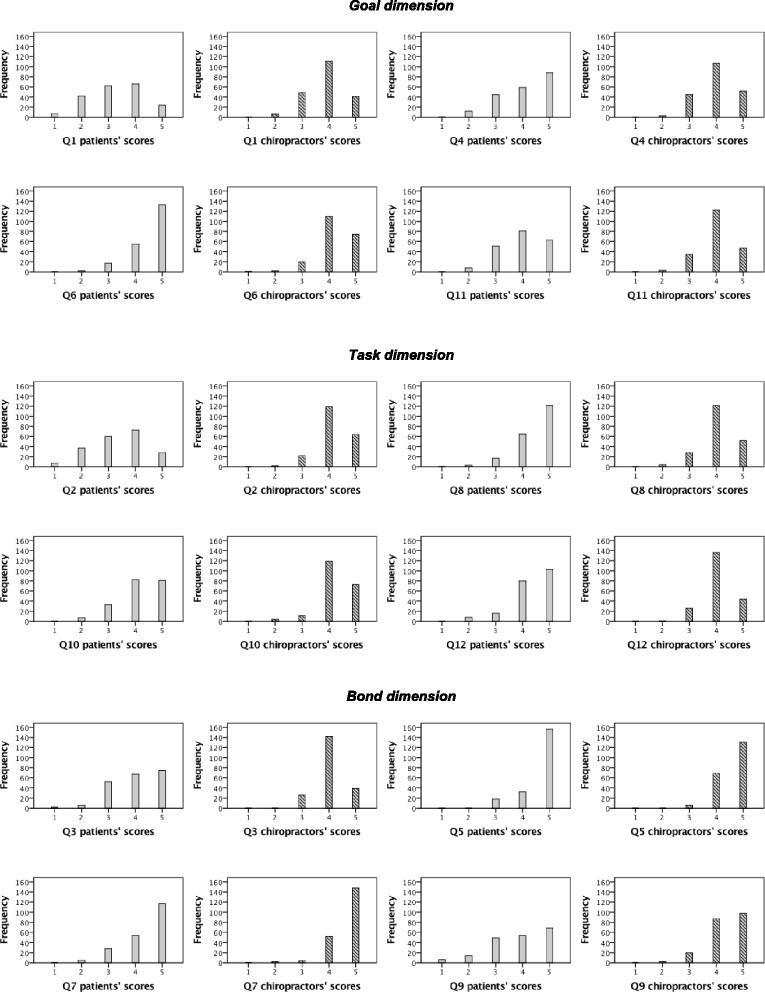
Frequencies of patients’ and chiropractors’ scores on individual questions of the WAV-12, grouped per dimension

Figure 2 presents frequencies of scores on individual questions of the WAV-12, grouped per dimension, both for patients and chiropractors. Although perceptions were rated very positive, patients’ ratings were more varied compared to the more skewed ratings of chiropractors.

### Agreement between patients’ and chiropractors’ scores

Results in Table 3 showed that there was poor agreement (ICC < 0.40) [35] between patients’ and chiropractors’ perspectives on the same working relationships.

**Table 3:** Agreement between patients’ and chiropractors’ perceptions on their working alliance

Patients’ and chiropractors’ perceptions	N	ICC^a^
Overall WAV-12	180	0.13
Goal dimension	196	0.07
Task dimension	199	0.14
Bond dimension	186	0.10

^a^ICC < 0.40 indicates poor agreement

### Influence of gender

Results in Tables 4 and 5 showed that there were no statistically significant differences in patients’ perceptions of their working relationships between being treated by a male or female chiropractor.

**Table 4:** Patients’ perceptions of the therapeutic alliance when treated by male and female chiropractors

Patients’ perceptions	Gender chiropractor	N	Mean^a^	SD	t(df)	*p*
Overall WAV-12	Male	86	4.09	±0.60	t(181) = −0.18	0.86
	Female	97	4.10	±0.59		
Goal dimension	Male	93	3.98	±0.66	t(195) = −0.11	0.92
	Female	104	3.99	±0.65		
Task dimension	Male	96	4.11	±0.59	t(199) = 0.19	0.85
	Female	105	4.09	±0.64		
Bond dimension	Male	90	4.17	±0.74	t(186) = −1.07	0.29
	Female	98	4.28	±0.66		

^a^Measured on a 1–5 Likert scale, 5 representing an optimal alliance

**Table 5:** Patient’s perceptions of the therapeutic alliance when treated by same sex and different sex chiropractors

Patients’ perceptions	Gender match	N	Mean^a^	SD	t(df)	*p*
Overall WAV-12	No match	78	4.11	±0.54	t(179) = 0.46	0.64
	Match	103	4.07	±0.63		
Goal dimension	No match	85	4.03	±0.62	t(193) = 1.03	0.30
	Match	110	3.93	±0.67		
Task dimension	No match	87	4.10	±0.60	t(196) = 0.14	0.89
	Match	111	4.09	±0.63		
Bond dimension	No match	81	4.20	±0.66	t(183) = −0.24	0.81
	Match	104	4.23	±0.73		

^a^Measured on a 1–5 point Likert scale, 5 representing an optimal alliance

### Influence of working experience

**Fig. 3:**
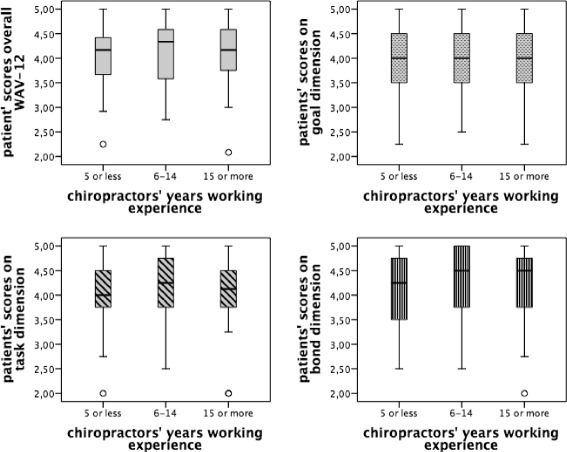
Patients’ perceptions in relation to years of experience of the chiropractor

The number of years of experience working as a chiropractor ranged from less than 1 to 31 years and was categorised into three similarly sized groups: 0–5 years (30.3 %), 6–14 years (36.8 %) and 15 years or more (32.9 %). The results in Fig. 3 showed that the differences in patients’ perceptions on their working relationship were minimal between these three experience categories and were not statistically significant (Table 6).

**Table 6:** Patients’ perceptions in relation to working experience of chiropractor

Patients’ perceptions	Years experience	N	Mean^a^	SD	F(df)	*p*
Overall WAV-12	0–5	59	4.04	±0.59	F(2) = 0.60	0.55
	6–14	65	4.15	±0.64		
	15+	59	4.09	±0.55		
Goal dimension	0–5	62	3.93	±0.66	F(2) = 0.38	0.68
	6–14	72	4.03	±0.69		
	15+	63	3.95	±0.65		
Task dimension	0–5	63	4.03	±0.61	F(2) = 0.58	0.56
	6–14	74	4.15	±0.66		
	15+	64	4.07	±0.55		
Bond dimension	0–5	60	4.15	±0.71	F(2) = 0.55	0.58
	6–14	67	4.28	±0.75		
	15+	61	4.25	±0.62		

^a^Measured on a 1–5 point Likert scale, 5 representing an optimal alliance

## Discussion

This study is the first to explore the quality of the therapeutic alliance between patients and chiropractors in The Netherlands as perceived by both patients and chiropractors. The measurement instrument used in this study assessed how both patients and chiropractors experienced collaboration in reaching agreement on treatment goals and treatment strategies to achieve their goals and how they perceived their affective bond. The results showed that, immediately after the third consultation, both patients and chiropractors perceived the quality of their working relationship overall to be very positive. Results on the quality of the three separate elements of the alliance showed that both patients and chiropractors slightly more often experienced the presence of the affective bond than collaboration in reaching consensus on treatment goals and treatment strategies. However, poor agreement was shown to exist between perceptions of patients and chiropractors of the quality of their alliance, both overall, and on the three elements of the alliance separately. Neither the chiropractors’ gender nor their years of experience, nor gender matching between chiropractor-patient pairs was shown to influence perceptions of patients on the quality of the alliance significantly.

Whereas the results of this study are encouraging in that patients felt that their interaction with their chiropractor was very good, they do not permit chiropractors to sit back and relax. The therapeutic alliance is a dynamic and developing process of collaboration between the patient and clinician and its strength often fluctuates during the course of care [2, 29]. Understanding how clinician and patient factors, such as their present mood, preoccupation with personal issues and severity of symptoms, as well as situational factors, such as excessive workload, lack of time, waiting time, and delays in improvement, may cause fluctuations in the strength of the therapeutic alliance over time is imperative for chiropractors. To maintain a positive alliance between the patient and the chiropractor, both parties have to demonstrate a commitment to collaborate during the course of the treatment for however long that takes [2, 29]. Important to note in this respect is that although previous research has confirmed a positive and consistent association between therapeutic alliance and clinical outcomes, the term ‘association’ does not indicate that there is a causal relationship between the two [36]. However, a recent study in the field of physical therapy and rehabilitation found a clear dose–response effect between the therapeutic alliance and clinical outcomes. Outcomes were better when interventions were combined with enhanced therapeutic alliance applications, compared to a limited application of factors that have been shown to enhance the therapeutic alliance [14].

The small differences between patients’ views and chiropractors’ views might disguise the fact that there was poor agreement between these perceptions. The results showed that chiropractors and patients had very different perceptions of the same working relationship. A lack of agreement between patient views and therapist views is consistent with the literature in psychotherapy. It was suggested that patient perceptions are more predictive for clinical outcomes compared to therapist perceptions, on the basis that patient perceptions were shown to remain more stable over time compared to therapists’ perceptions [4, 6, 29]. However, no studies were conducted to test this hypothesis.

One could argue that the poor agreement between patients’ and chiropractors’ views is the most important finding of this study for clinical practice. Chiropractors should be aware of this possible dissonance and should verify their views with the patient, especially concerning views on setting treatment goals and treatment strategies. Dissonance in agreement on treatment goals and treatment strategies can jeopardise patient management, especially in the case of chronic disease, where self-management and self-efficacy from patients are essential [26].

Somewhat surprisingly, the results in the present study did not demonstrate any difference between the varying years of experience as a chiropractor on patients’ perceptions of the quality of any of the elements of the therapeutic alliance. In a study in psychotherapy, which used the same version of the WAV-12 as the present study [29], interactions with respect to agreement on treatment strategies with therapists with over 20 years of experience were perceived significantly less positive compared to interactions with less experienced therapists (10–19 years). Besides a possible difference in education, it was postulated by the authors that a more experienced therapist may more frequently work on ‘an automatic pilot’, and spent less time and energy on discussing treatment strategies as to how to achieve the treatment goals with their patients. Working on the ‘automatic pilot’ may also be true for more experienced chiropractors. At the same time, as a result of patients being informed better and having their own ideas about health, experienced chiropractors may have consciously or unconsciously adapted their patient management to accommodate for more collaboration, thereby improving the therapeutic alliance.

Studies on empathy and communication in relation to the therapeutic alliance [24, 37] found that female physicians displayed more empathy and active collaboration than male physicians, both of which behaviours are considered to improve the strength of the alliance. Although there might be differences in displaying empathy and active collaboration between male and female chiropractors, this study showed that patients’ perceptions of the quality of the alliance did not differ for male and female chiropractors. This lack of difference might partly be explained by the fact that chiropractic care is considered as alternative medicine in The Netherlands and is still fairly unknown to many patients. Chiropractors will have to make an effort to explain and promote their care and might, therefore, be more motivated to engage and collaborate with the patient, independent of their gender. Educational differences between medicine and chiropractic might play a role as well. Historically, chiropractic has always advocated a holistic approach, in which patients are attended to in their whole identity as opposed to the former disease-centred approach in medicine. Chiropractors might, therefore, be more experienced in practising patient-centred care, which involves empathic and collaborative behaviour. Their training may thus diminish any gender differences in displaying empathy that was seen amongst male and female physicians. Furthermore, many chiropractors in The Netherlands decided to become a chiropractor because of their personal experience with chiropractic as a patient. It has been suggested that to improve empathic behaviour a clinician should be a patient himself [18]. Having started as a patient may be an advantage for many chiropractors, both female and male, in displaying empathy.

Some of these reasons could also explain the unexpected lack of difference in patients’ perceptions of the therapeutic alliance between gender-matched and unmatched chiropractor-patient pairs. Other explanations for this lack of difference might be that patients who have a clear preference for a female or male chiropractor can often be booked with the chiropractor of their choice since many chiropractic clinics have both female and male chiropractors working in the clinic. Consulting a chiropractor of the gender of their preference may give patients a more positive stance in the working relationship. Recommendation by an acquaintance or family member might also positively influence the patient’s stance in the working relationship at the start of the treatment, irrespective of factors such as gender or age of the chiropractor. Although this could be pure coincidence, the freedom of the patient to choose a chiropractor of the gender of preference may explain why almost two-thirds of the analysed working relationships in this study involved gender-matched chiropractor-patient pairs.

There are several limitations to this study, which potentially compromise the external validity of this study. Firstly, participation in this study was on a voluntary basis. Chiropractors who advocate patient-centred care and have good communication skills could be overrepresented in this study. Secondly, questionnaires were sent to the participating chiropractors in advance, which allowed them to read the questions in advance, and as a result, allowed them to give their best performance to the patient with respect to the alliance. Thirdly, perceptions were observed at one moment in time and were, therefore, influenced by the mood of the participant at that particular moment. In addition, patients who did not trust their chiropractor not to open the envelope and read their perceptions may have reported more positive perceptions. Furthermore, since all questions were scored in the same direction, participants may have failed to pay close attention to the questions and may have answered all items in the same way. The use of a questionnaire, which was developed in the field of psychotherapy, might be a further limitation. In the absence of a better alternative, the Working Alliance Inventory (WAI) has been proven valid and usable in physiotherapy settings [32]. Nevertheless, participants in the present study found some questions confusing or ambiguous. Noteworthy in this respect is that more than 10 % of patients did not complete all questions as requested. Particularly issues related to the quality of the affective bond between the patient and chiropractor were left unanswered. Although no feedback was requested from the participants, several patients reported questions exploring the quality of the affective bond to be irrelevant or inappropriate. And even though it could be argued that these questions were not relevant, some questions are considered less applicable in a chiropractic setting. Similar responses were found in a physiotherapy setting, and the authors suggested to develop a more conceptually sound measure of the therapeutic alliance for use in physiotherapy [32]. Furthermore, several chiropractors commented on the choice of response scale. The current options (seldom to always) were found inappropriate and not applicable to rating perceptions, and suggestions were made using options as to how much the participant agreed with each statement (strongly disagree to strongly agree). This modification was applied in a study on perceptions of the quality of the therapeutic alliance in a primary care setting, along with some modifications in the phrasing of the statements, and proved useful [10]. As in any observational study, confounders could not be fully controlled for in this study. Specific patient or chiropractor characteristics could have influenced the results. Some patients might have had severe symptoms, unintentionally influencing their perceptions more negatively. Female and male chiropractors might have treated patients with different levels of symptoms, different levels of cognitive ability, and of different ages, which could all have influenced perceptions on gender and experience. These differences, however, reflect daily practice and were, therefore, considered acceptable.

One important recommendation for further research is to develop a uniform instrument to assess the quality of the therapeutic alliance, with an appropriate response scale that could be used in primary care settings. This uniform instrument should be translated and validated for use in different countries. The second recommendation for research is to further investigate the level of agreement on treatment goals and treatment strategies to achieve these goals between patients and chiropractors. In the present study, only perceptions of collaboration in reaching agreement were studied. It would be useful for clinical practice to conduct a study examining to what extent patients and chiropractors agree on treatment goals and treatment strategies, as was conducted for patients with diabetes and their physicians and showed poor levels of agreement [26].

## Conclusion

The results of this study showed that both patients and chiropractors perceived their working alliances very positive. Contrary to what was expected, no significant differences were shown to exist in patients’ perceptions in relation to the chiropractors’ gender or years of working experience.

The most important finding with respect to clinical practice was that poor agreement was found between the perceptions of patients and chiropractors on the same working relationship. This dissonance in perceptions must be given serious consideration by chiropractors. In order to develop, improve and maintain a positive therapeutic alliance, chiropractors should, during the course of treatments, continue to collaborate with the patient and frequently verify their perception with the views of the patient, especially with respect to determining treatment goals and treatment strategies to achieve these goals.

### Ethics approval and consent to participate

Ethics approval was applied for and granted by the Anglo-European College of Chiropractic (AECC) Research Ethics Subcommittee. All questionnaires were completed anonymously and consent to participate was implied by completion of the questionnaire in which an explanation of the study was given.

### Consent for publication

Not applicable.

### Availability of data and materials

The dataset supporting the conclusions of this article is included in an additional file.

## Additional file

Additional file 1: Literature search and search strategy. (DOCX 96 kb)

## Abbreviations

WAI: working alliance inventory
WAV: Werkalliantie Vragenlijst (Validated translation into Dutch from WAI)

## References

[1] Mead N, Bower P (2002). Patient-centred consultations and outcomes in primary care: a review of the literature. Patient Educ Counsel.

[2] Horvath AO, Del Re AC, Flückiger C, Symonds D (2011). Alliance in individual psychotherapy. Psychotherapy (Chic).

[3] Horvath AO, Symonds BD (1991). Relation between working alliance and outcome in psychotherapy: A meta-analysis. J Couns Psychol.

[4] Martin DJ, Garske JP, Davis MK (2000). Relation of the therapeutic alliance with outcome and other variables: A meta-analytic review. J Consult Clin Psychol.

[5] Bordin ES (1979). The generalizability of the psychoanalytic concept of the working alliance. Psychotherapy Theory Res Practice.

[6] Ardito RB, Rabellino D (2011). Therapeutic alliance and outcome of psychotherapy: historical excursus, measurements, and prospects for research. Frontiers Psychol.

[7] Hatcher RL, Gillaspy JA (2006). Development and validation of a revised short version of the Working Alliance Inventory. Psychother Res.

[8] Arbuthnott A, Sharpe D (2009). The effect of physician-patient collaboration on patient adherence in non-psychiatric medicine. Patient Educ Couns.

[9] Derksen F, Bensing J, Lagro-Janssen A (2013). Effectiveness of empathy in general practice: a systematic review. Bri J General Practice J Royal College General Practitioners.

[10] Fuertes JN, Mislowack A, Bennett J, Paul L, Gilbert TC, Fontan G, Boylan LS (2007). The physician-patient working alliance. Patient Educ Couns.

[11] Pinto RZ, Ferreira ML, Oliveira VC, Franco MR, Adams R, Maher CG, Ferreira PH (2012). Patient-centred communication is associated with positive therapeutic alliance: a systematic review. J Physiotherapy.

[12] Rolfe A, Cash-Gibson L, Car J, Sheikh A, McKinstry B (2014). Interventions for improving patients’ trust in doctors and groups of doctors. Cochrane Database Syst Rev.

[13] Ferreira PH, Ferreira ML, Maher CG, Refshauge KM, Latimer J, Adams RD (2013). The therapeutic alliance between clinicians and patients predicts outcome in chronic low back pain. Phys Ther.

[14] Fuentes J, Armijo-Olivo S, Funabashi M, Miciak M, Dick B, Warren S, Rashiq S, Magee DJ, Gross DP (2014). Enhanced Therapeutic Alliance Modulates Pain Intensity and Muscle Pain Sensitivity in Patients With Chronic Low Back Pain: An Experimental Controlled Study. Phys Ther.

[15] Hall AM, Ferreira PH, Maher CG, Latimer J, Ferreira ML (2010). The influence of the therapist-patient relationship on treatment outcome in physical rehabilitation: a systematic review. Phys Ther.

[16] Hojat M, Louis DZ, Markham FW, Wender R, Rabinowitz C, Gonnella JS (2011). Physicians’ empathy and clinical outcomes for diabetic patients. Acad Med J Assoc Am Med Colleges.

[17] Jani BD, Blane DN, Mercer SW (2012). The role of empathy in therapy and the physician-patient relationship. Forschende Komplementarmedizin.

[18] Neumann M, Bensing J, Mercer S, Ernstmann N, Ommen O, Pfaff H (2009). Analyzing the “nature” and “specific effectiveness” of clinical empathy: a theoretical overview and contribution towards a theory-based research agenda. Patient Educ Couns.

[19] Norfolk T, Birdi K, Walsh D (2007). The role of empathy in establishing rapport in the consultation: a new model. Med Educ.

[20] Hall MA, Zheng B, Dugan E, Camacho F, Kidd KE, Mishra A, Balkrishnan R (2002). Measuring patients’ trust in their primary care providers. Med Care Res Rev.

[21] Tarrant C, Stokes T, Baker R (2003). Factors associated with patients’ trust in their general practitioner: a cross-sectional survey. Bri J General Practice J Royal College General Practitioners.

[22] Thom DH (2001). Physician behaviors that predict patient trust. J Fam Pract.

[23] Roter DL, Hall JA (1998). Choices: biomedical ethics and women’s health. Why physician gender matters in shaping the physician-patient relationship. J Women’s Health.

[24] Roter DL, Hall JA, Aoki Y (2002). Physician gender effects in medical communication: A meta-analytic review. J Am Med Assoc.

[25] Jahng KH, Martin LR, Golin CE, DiMatteo MR (2005). Preferences for medical collaboration: Patient-physician congruence and patient outcomes. Patient Educ Couns.

[26] Heisler M, Vijan S, Ubel PA, Bernstein SJ, Hofer TP, Anderson RM (2003). When Do Patients and Their Physicians Agree on Diabetes Treatment Goals and Strategies, and What Difference Does It Make?. J Gen Intern Med.

[27] Dahm MR (2012). Tales of Time, Terms, and Patient Information-Seeking Behavior - An Exploratory Qualitative Study. Health Commun.

[28] Levinson W, Gorawara-Bhat R, Lamb J (2000). A study of patient clues and physician responses in primary care and surgical settings. J Am Med Assoc.

[29] Stinckens N, Ulburghs A, Claes L (2009). De werkalliantie als sleutelelement in het therapiegebeuren. Meting met behulp van de WAV-12, de Nederlandstalige verkorte versie van de Working Alliance Inventory. Tijdschrift Klinische Psychologie.

[30] Horvath AO, Greenberg LS (1989). Development and validation of the Working Alliance Inventory. J Couns Psychol.

[31] Tracey TJ, Kokotovic AM (1989). Factor structure of the Working Alliance Inventory. Psychol Assess J Consult Clin Psychol.

[32] Besley J, Kayes NM, McPherson KM (2011). Assessing the measurement properties of two commonly used measures of therapeutic relationship in physiotherapy. N Z J Physiother.

[33] Elvins R, Green J (2008). The conceptualization and measurement of therapeutic alliance: an empirical review. Clin Psychol Rev.

[34] Miller SEKM (2011). An Examination of Therapeutic Alliance in Chinese Medicine. Aust J Acupuncture Chinese Med.

[35] Cicchetti DV (1994). Guidelines, criteria, and rules of thumb for evaluating normed and standardized assessment instruments in psychology. Psychol Assess.

[36] Lucas RM, McMichael AJ (2005). Association or causation: evaluating links between “environment and disease”. Bull World Health Organ.

[37] Hojat M, Gonnella JS, Nasca TJ, Mangione S, Vergare M, Magee M (2002). Physician empathy: definition, components, measurement, and relationship to gender and specialty. Am J Psychiatr.

