# Supplementary material for: Perceptions of the quality of the therapeutic alliance in chiropractic care in The Netherlands: a cross-sectional survey
Source: Chiropr Man Therap. 2016 Jun 8;24:18. doi: 10.1186/s12998-016-0100-4 (PMC4897913; doi:10.1186/s12998-016-0100-4)
Supplement: Additional file 1: — Literature search and search strategy. (DOCX 96 kb) [file 12998_2016_100_MOESM1_ESM.docx]

**Appendix 1: Literature search and search strategy.**

To identify and review relevant literature the following seven electronic databases were searched from 1990 to May 2014: Medline, Cochrane Library, PsycINFO, CINAHL, Index to Chiropractic Literature, Allied and Complementary Medicine Database and Alt Health Watch. Key search terms included: therapeutic alliance (Title/Abstract), therapeutic relationship (Title/Abstract), working alliance (Title/Abstract), rapport (Title/Abstract), and physician-patient relations (MeSH term/Subject term) or professional-patient relations (MeSH term/Subject term). The Subject term ‘Interpersonal Relations’ was used in the Alt Health Watch search. Reference lists of relevant publications were manually screened for further citations.

The inclusion criteria were any research focusing on identifying, exploring or discussing components or aspects of the therapeutic alliance/therapeutic relationship/working alliance/rapport between a doctor or health care professional and individual adult patients in face-to-face interactions in primary care settings. All papers had to be available in English or Dutch and in full text.

Exclusion criteria were any research on the topic of the therapeutic alliance focusing specifically on mental health care settings, psychoanalysis, psychotherapy, mental or psychiatric disorders (MeSH terms), eating disorders, substance misuse, sexual abuse, HIV, and communication through interpreters. The reason for narrowing the scope of this review by excluding articles specifically on mental or psychiatric disorders (MeSH terms), eating disorders, substance misuse, sexual abuse and HIV was practical. Reviewing all these articles would be too time-consuming and often patients consulting with these problems are at some stage referred out to secondary or mental care, both being outside the scope of the review.

The selection process is shown in figure 3.
